# Computer-aided design/computer-aided manufacturing of hydroxyapatite scaffolds for bone reconstruction in jawbone atrophy: a systematic review and case report

**DOI:** 10.1186/s40902-015-0048-7

**Published:** 2016-01-04

**Authors:** Umberto Garagiola, Roberto Grigolato, Rossano Soldo, Marco Bacchini, Gianluca Bassi, Rachele Roncucci, Sandro De Nardi

**Affiliations:** 1 Biomedical Surgical and Dental Sciences Department, Maxillo-Facial and Odontostomatology Unit, Fondazione Cà Granda IRCCS Ospedale Maggiore Policlinico, University of Milan, Milan, Italy; 2 Private Practice, Milan, Italy

**Keywords:** Computer-aided design/computer-aided manufacturing, Hydroxyapatite, Scaffold, Review

## Abstract

**Background:**

We reviewed the biological and mechanical properties of porous hydroxyapatite (HA) compared to other synthetic materials. Computer-aided design/computer-aided manufacturing (CAD/CAM) was also evaluated to estimate its efficacy with clinical and radiological assessments.

**Method:**

A systematic search of the electronic literature database of the National Library of Medicine (PubMed-MEDLINE) was performed for articles published in English between January 1985 and September 2013. The inclusion criteria were (1) histological evaluation of the biocompatibility and osteoconductivity of porous HA in vivo and in vitro, (2) evaluation of the mechanical properties of HA in relation to its porosity, (3) comparison of the biological and mechanical properties between several biomaterials, and (4) clinical and radiological evaluation of the precision of CAD/CAM techniques.

**Results:**

HA had excellent osteoconductivity and biocompatibility in vitro and in vivo compared to other biomaterials. HA grafts are suitable for milling and finishing, depending on the design. In computed tomography, porous HA is a more resorbable and more osteoconductive material than dense HA; however, its strength decreases exponentially with an increase in porosity.

**Conclusions:**

Mechanical tests showed that HA scaffolds with pore diameters ranging from 400 to 1200 μm had compressive moduli and strength within the range of the human craniofacial trabecular bone. In conclusion, using CAD/CAM techniques for preparing HA scaffolds may increase graft stability and reduce surgical operating time.

## Background

Bone grafting is a surgical procedure that replaces a missing bone with material from the patient’s own body, using an artificial, synthetic, or natural substitute. Large bone defects and poor bone healing require augmentation to facilitate new bone formation. Long-term prognosis is adversely affected by inadequate bone volume; thus, an adequate three-dimensional amount of a bone at the site of implant placement is essential for successful implant therapy.

Bone formation after grafting is characterized by three types of bone growth: osteogenesis, osteoinduction, and osteoconduction. Osteogenesis occurs when vital osteoblasts originate from the bone graft material and contribute to new bone growth. Osteoinduction involves the stimulation of osteoprogenitor cells to differentiate into osteoblasts to begin new bone formation. Osteoconduction occurs when the bone graft material serves as a scaffold for new bone growth that is perpetuated by the native bone. Osteoblasts from the margin of the defect that are being grafted use the bone graft material as a framework to spread and generate a new bone. A bone graft material is osteoconductive and osteoinductive and will not only serve as a scaffold for currently existing osteoblasts but will also trigger the formation of new osteoblasts, thereby, at least theoretically, promoting faster integration of the graft.

Numerous methods have been used in guided bone regeneration (GBR) [1, 2]. One of the most common techniques involves harvesting and implantating fresh autogenous bone grafts taken from the same patient. However, this is an expensive procedure that requires hospitalization and has a potential risk of donor site morbidity. To avoid such complications, clinicians have developed the use of biomaterials as substitutes for alveolar bones. Other types of grafts available for the maxilla and mandible include allogeneic, alloplastic, and xenogeneic ones. Autografts are the only grafts that provide osteoinductivity; unfortunately, autografts often have unpredictable resorption, morbidity at the donor site, and limited bone availability, which has stimulated research to find new alternatives for bone tissue engineering.

Maiorana et al. [3] compared the healing of onlay block bone grafts with deproteinized bovine bone material (DBBM) coverage and the healing of grafts without such coverage, with the goal of clinically evaluating the ability of DBBM to reduce grafted bone resorption. The results indicated that DBBM can be placed over grafted areas, taking advantage of its osteoconductive properties and compensating for the natural bone resorption caused by remodeling.

The main advantage of alloplastic materials is that, because of their completely synthetic composition, there is no risk of pathogen transmission. Moreover, synthetic materials allow control of all features (e.g., chemical composition, dimensions and interconnectivity of the macropores, specific morphology of blocks and granules) to adapt the material to the specific clinical situation. Currently, there are three different groups of allogeneic material used in bone regeneration: calcium phosphate, bioglass, and polymers. Of these, calcium phosphate, and in particular hydroxyapatite (HA), and β-tricalcium phosphate (β-TCP) are the most studied materials because of their similar composition to inorganic bones. In a study about sinus elevation, Szabò et al. [4] concluded that the grafting of β-TCP was followed by the formation of new bone of similar quality and quantity to that observed after grafting with autogenous bone. Comparisons in other studies have shown that β-TCP is a satisfactory graft material, even without autogenous bone, and a second operation is not necessary, so donor site morbidity can be avoided using this material.

Suba et al. [5, 6] compared the effects of β-TCP and an autogenous bone graft. β-TCP proved to be an effective bone replacement material with osteoconductivity and was capable of gradual disintegration, providing space for the regenerating bone. The new bone density was similar on both sides, with no significant difference. After 6 months, insertion of the β-TCP graft resulted in formation of a stable bony bed that was suitable for the anchoring of dental implants. In a prospective human study, Simunek et al. [7] compared 48 sinus grafting operations using β-TCP, DBBM (pure or mixed with 10 to 20 % autogenous bone), and autogenous bone. When autogenous bone was used, 49.2 ± 3.1 % of new bone was found, which was significantly higher than in all of the other groups. A higher proportion (34.2 ± 13.1 %) of new vital bone was found in the DBBM group compared to the β-TCP (21.4 ± 8.1 %) and β-TCP composite graft groups (24.0 ± 6.6 %; *p* < 0.05). No significant difference was found between single-component grafts and the corresponding composite grafts. Sinus augmentation with these augmentation materials is a well-accepted procedure; however, autogenous bone alone was the best material. More new bone was found using DBBM than β-TCP. The addition of 10–20 % autogenous bone to the bone substitute did not significantly affect new bone formation.

## Review

**Fig. 1:**
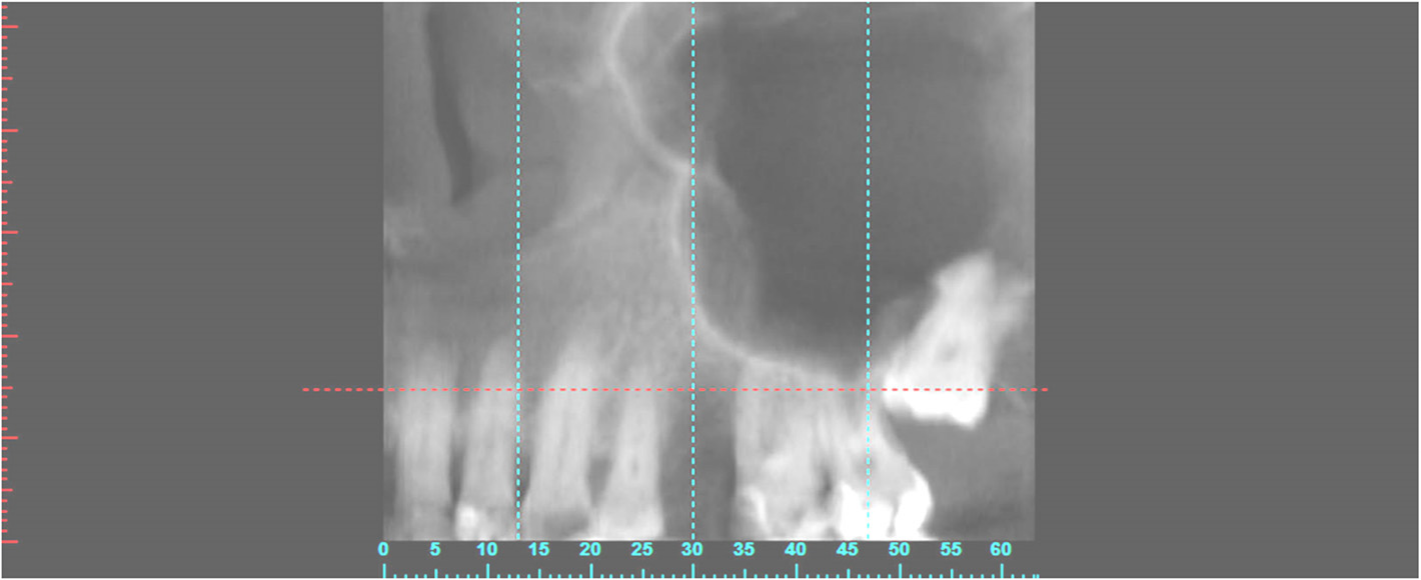
CBCT with bone defect

**Fig. 2:**
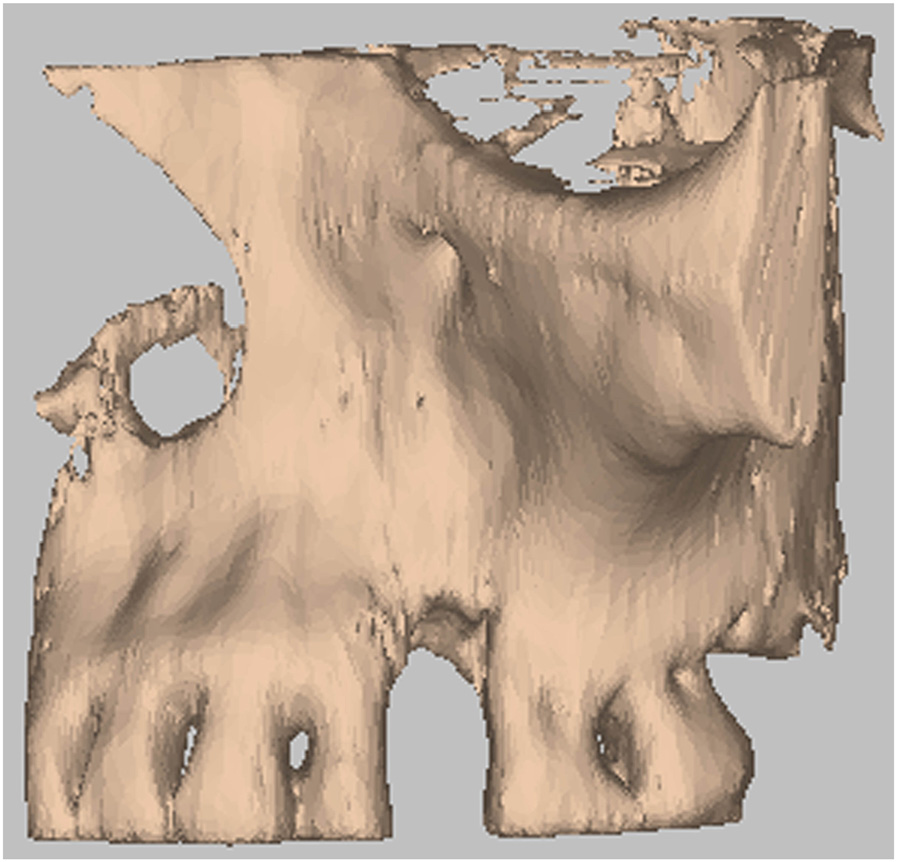
3D volumetric reconstruction of the bone defect

**Fig. 3:**
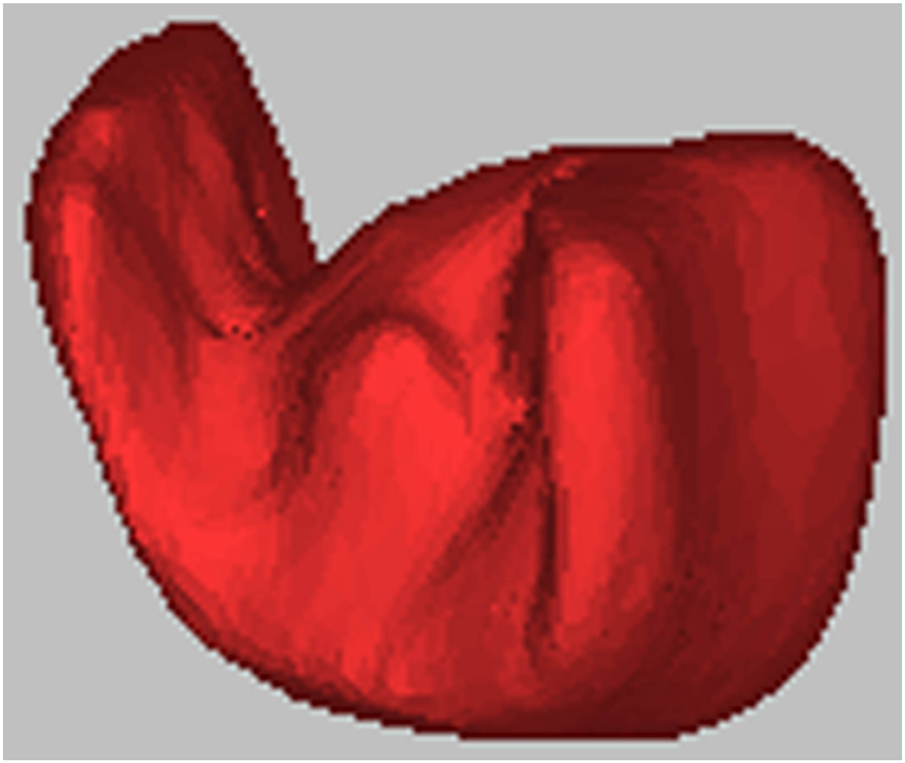
Hydroxyapatite bone graft, built using CAD/CAM

**Fig. 4:**
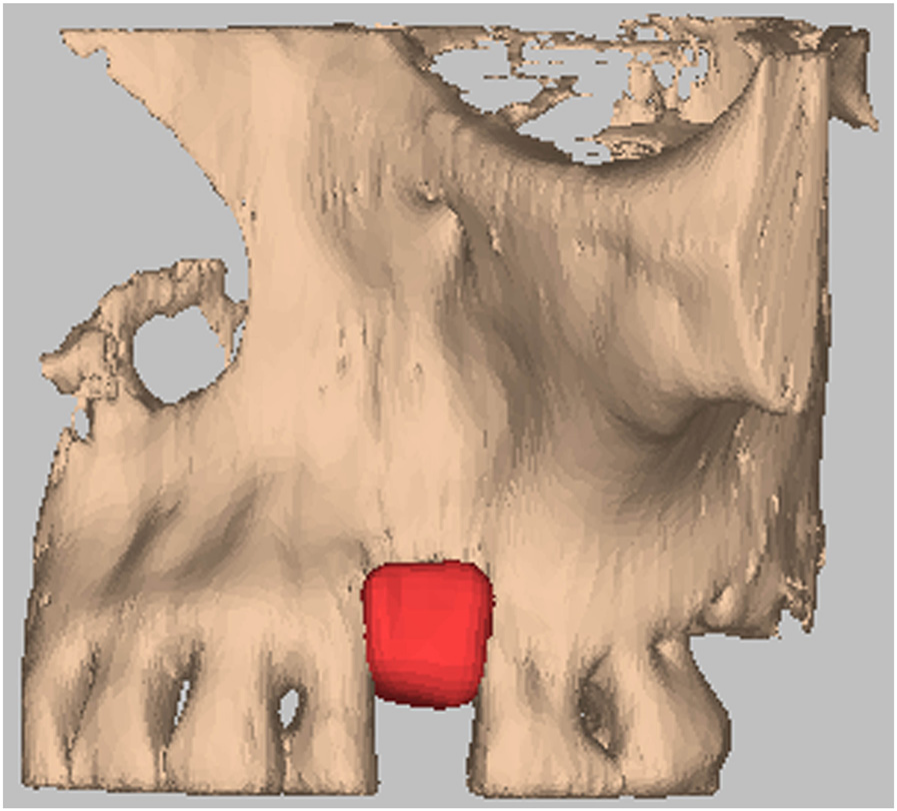
Hydroxyapatite bone graft CAD/CAM inserted at the level of the bone defect

The aim of the review was first to confirm the optimal mechanical and biological properties of HA in vitro and in vivo compared to other synthetic materials. Second, a computer-aided design/computer-aided manufacturing (CAD/CAM) technique using a porous HA scaffold was evaluated to estimate its efficacy with clinical and radiological assessments (Figs. 1, 2, 3, and 4).

A systematic search of the electronic literature database of the National Library of Medicine (PubMed-MEDLINE) was conducted using search terms to identify relevant articles regarding the aim of the review. The search was restricted to articles published in English between January 1985 and September 2013, and the references of the retrieved articles were also searched.

Inclusion criteria were (1) histological evaluation of the effective biocompatibility and osteoconductivity of porous HA in vivo and in vitro, (2) evaluation of the mechanical properties of HA in relation to its porosity, (3) comparative evaluation of biological and mechanical properties between several biomaterials to assess whether HA was appropriate in different clinical situations, and (4) a clinical and radiological evaluation of the precision of CAD/CAM techniques. A clinical case was also reported to illustrate the use of the CAD/CAM technique.

### Hydroxyapatite

HA is considered osteoconductive and non-resorbable, whereas β-TCP is osteoconductive but is resorbed quickly [8–11], because calcium and phosphate ions released from β-TCP during degradation are used to form new tissue, and the resorption of β-TCP provides space for new bone formation. Usually, in small defects, bone healing is much quicker using β-TCP than using HA; in contrast, in larger defects, β-TCP resorption is too rapid and the resulting space cannot be sufficiently filled by a new bone quickly [12]. For this reason, combinations of HA and β-TCP have been studied to adapt the material to different clinical situations [13]. Recently, some studies evaluated the possibility of using zirconia as a scaffold, but the results were not as good as those with HA scaffolds.

HA, Ca_10_(PO_4_)_6_(OH)_2_, is a calcium phosphate ceramic that is currently used for bone tissue repair in non-load-bearing applications. It has high biocompatibility and osteoconductivity and a strong capacity to bind both hard and soft tissues. Clinical indications for the use of this material are due to the chemical and physical characteristics of the grafts. It is available in a porous (micro or macro) or non-porous form, resorbable or non-resorbable form, and blocks or particles. The chemical properties depend on the calcium to phosphate ratio, the pH of the material, the ionic substitution, and elementary impurities [14]. The crystal nature of HA, especially its dimensions, is responsible for whether it is resorbable or not [15]. The porosity determines the extent of blood penetration and blood vessel proliferation in the graft. Pore dimensions of 250–350 μm are considered ideal; however, its strength decreases exponentially with increased porosity [16–19]. Solid blocks have high resistance to compression but are fragile and can migrate during the healing process and cannot be used in load-bearing situations. As cells infiltrate the scaffold and proliferate, the scaffold degrades, providing more space for continued cell growth and tissue formation. Eventually, the scaffolds are partially resorbed and incorporated into adjacent and remodeled bone. In vivo degradation of HA occurs by aqueous dissolution in body fluids, resorption by osteoclasts and multinuclear cells, and phagocytosis of particles by macrophages. As HA scaffolds degrade, strength is lost progressively through mass loss. For load-bearing applications, ingrown bone tissue must provide compensating strength to support the mechanical load at the site of implantation. Usually, HA is mixed with autologous bone to increase the graft volume with an osteoconductive material. An issue with HA use is the difficulty of implant placement; thus, HA is usually combined with calcium sulfate (CaSO_4_), which is completely resorbable. In association with HA, CaSO_4_ prevents the early loss of HA particles, avoiding soft tissue invasion during the first month [20].

A study by Piattelli et al. in 1994 [21] was the first to investigate HA graft biocompatibility in vivo. In total, 20 HA-coated implants were placed in rabbit femurs and evaluated after 6 months. The data were analyzed by laser scanning microscopy (LSM). In all specimens, there was intimate contact between the bone and HA; in some portions, mineralized bone was in tight, direct contact with the HA, while in others, a basophilic and apparently unmineralized material was present between the bone and HA. LSM showed fluorescence at many areas of the interface, in osteocyte lacunae, and inside the coating. Organic bonding between the bone and HA was hypothesized. Since then, many studies have compared biological and mechanical properties of HA in vivo to other biomaterials used for grafts in regenerative surgery.

The study by Hollister et al. in 2005 [22] analyzed synthetic scaffolds to evaluate whether their mechanical properties were in the range of craniofacial tissue and could support bone regeneration for craniofacial reconstruction. The authors evaluated whether the designed scaffold architecture could achieve the desired elasticity and permeability and whether the designed external shape could match the craniofacial anatomy. These scaffolds could be fabricated from a wide range of biomaterials, including titanium, degradable polymers, and degradable calcium phosphate ceramics. Mechanical tests showed that fabricated scaffolds had compressive moduli ranging from 50 to 2900 MPa and compressive strength ranging from 2 to >56 MPa, within the range of the human craniofacial trabecular bone. In vivo testing of the designed scaffolds showed that they could support bone regeneration via delivery of BMP-7, carried by human gingival fibroblasts, in a mouse model. Designed HA scaffolds with pore diameters ranging from 400 to 1200 μm were implanted in minipig mandibular defects for 6 and 18 weeks. The results showed substantial bone ingrowth (40–50 % at 6 weeks and 70–80 % at 18 weeks) for all scaffolds, with no significant difference based on pore diameter or material.

The aim of the study by Malmström et al. in 2007 [23] was to evaluate the effects of material composition and surface topography on bone ingrowth and bone contact. Macroporous ceramic scaffolds designed in zirconia (ZrO_2_) and HA with identical macroporosity were used. The scaffolds were implanted in the rabbit tibia (cortical bone) and femur (trabecular bone). After 6 weeks of implantation, the tissue response was assessed with histology and histomorphometry. The results showed significantly more bone ingrowth and bone contact in the HA than in the zirconia scaffolds. Surface topography had no significant effect on bone contact inside the macropores regardless of material. This was observed in both cortical and trabecular bone sites. This study suggests that the difference between HA and zirconia was due to the difference in material chemistry.

In another study in 2009 [24], scaffolds of ZrO_2_ and HA were placed in the maxilla of 12 subjects using a randomization protocol. After 3 months of healing, biopsies were harvested, comprising the scaffolds and the surrounding bone tissue. The biopsies were processed for histological evaluations and morphometric analyses (bone ingrowth and bone-to-scaffold contact). Healing was uneventful in all cases. All of the scaffolds demonstrated a measurable bone response using light microscopy and scanning electron microscopy (SEM). Microporous HA scaffolds revealed four times more bone ingrowth and seven times higher bone contact area compared to the ZrO_2_ scaffolds. The results showed that the chemistry and microporosity of HA promoted more bone ingrowth and bone contact than those of ZrO_2_ scaffolds in the human maxilla.

In 2009, Park et al. [25] compared a new bone graft substitute (N-HA) derived from a hen eggshell alone or in combination with calcium sulfate (CS) with a commercial bone substitute, anorganic bovine bone (Bio-Oss). Critical size defects were created in the calvaria of 56 rats. Animals were divided into four groups and treated with (1) unfilled defects, (2) N-HA grafts, (3) Bio-Oss grafts, and (4) N-HA/CS grafts. The percentage of newly formed bone (NB%) was evaluated histomorphometrically after 6 and 12 weeks. The N-HA group showed more new bone formation than the other groups at 6 and 12 weeks.

In 2010, Warnke et al. [26] evaluated HA and β-TCP biocompatibility in vitro using CAD scaffolds. In this study, the behavior of human osteoblasts on HA and β-TCP scaffolds was investigated. The commonly used bone replacement material, Bio-Oss, was used as a control. Biocompatibility was assessed by SEM; fluorescence microscopy after staining for cell vitality with fluorescein diacetate (FDA) and propidium iodide (PI); and the MTT, LDH, and WST biocompatibility tests. Both versions were colonized by human osteoblasts; however, more cells were seen on HA than on β-TCP scaffolds. Cell vitality staining and MTT, LDH, and WST tests showed superior biocompatibility of HA scaffolds to Bio-Oss, whereas Bio-Oss was more biocompatible than β-TCP.

### Computer-aided design/computer-aided manufacturing

The recent study by Kwon et al. [27] evaluated in vitro (cell attachment test) and in vivo (implantation test on the rabbit tibia) biocompatibility of solid freeform fabrication (SFF) and polymer replication method (PRM) HA scaffolds with 45 % porosity. The results showed that HA scaffolds fabricated by SFF had no adverse effects in vitro, such as cytotoxicity and hemolysis, or in vivo, such as irritation and sensitization. The SFF and PRM types were similar in interfacial strength. However, HA scaffolds fabricated by SFF are a relatively new technology used to produce objects of complex shapes directly from CAD files. The average maximum load of the SFF type (169 N) was higher than that of the PRM type (153 N). This means that a SFF-type implant would have a comparative advantage over conventional scaffolds in terms of osteoconduction. It was found that biological interactions might increase through the use of porous bone scaffolds fabricated by SFF. The results of microscopic examinations showed no pathological reaction in the graft or surrounding tissue. All of the scaffold groups showed a good healing response with no adverse tissue reactions in the HA scaffold fabricated by SFF and PRM after 12 weeks. Thus, HA scaffolds fabricated by SFF and PRM were biocompatible and osteoconductive.

Mangano et al. [28] investigated the use of CAD/CAM HA scaffold on sinus augmentation and vertical ridge augmentation of the mandible and maxilla in association with the position of titanium implants, reporting excellent clinical results. In the first study, the authors evaluated whether the anatomically shaped, custom-made scaffolds matched the shape of the bone defects in the maxilla of 10 patients. From the histomorphometric measurements of bone cores, retrieved 8 months after augmentation, new bone was available clinically to allow correct implant placement. In total, 10 implants were positioned. At the end of the study, no implant was lost and all implants were functioning, for an overall survival rate of 100 %. Caused into showed, suppuration, and exudation; was clinically mobile; or showed continuous peri-implant radiolucency, and no prosthetic complication was reported.

In a second study, Figliuzzi et al. [29] observed custom-made HA scaffolds to augment the posterior mandibular bone and minimize surgery when severe atrophy was present. No clinical complication was observed during the 6-month healing period. At the 6-month recall, clinically available, newly formed, well-integrated bone completely filled the mandibular posterior defects. The specimens were made of preexisting, compact mature bone undergoing remodeling, newly formed trabecular bone, and some biomaterial particles. The bone was well organized, with several osteons in evidence. Inside the porous HA structure, new bone formation was observed, with newly formed osteoid matrix undergoing mineralization. Implants were placed. At the 1-year follow-up, the implant-supported prosthetic restorations showed a good functional and esthetic integration. The reliability of custom-made HA scaffolds was also evaluated in five patients undergoing 10 maxillary sinus augmentations in association with a PTFE guide [30]. The clinically sized, anatomically shaped custom-made HA blocks fitted securely into the sinus. No surgical complication occurred. Immediately after surgery, post-operative intraoral periapical radiographs showed that the HA blocks precisely filled the defects. At 6 months after surgery, radiographic CBCT analysis confirmed post-grafting opacity of the maxillary sinus floor in all patients. No clinical sign of sinus pathology was observed, and no patient showed any sign of maxillary sinusitis. In total, 19 implants were placed. At the end of the study, after 2 years of functional loading, all of the implants were functional, and no clinical or prosthetic complications occurred. The radiographic evaluation revealed a low tendency for marginal bone resorption and good stability of peri-implant bone tissue.

### Clinical case

**Fig. 5:**
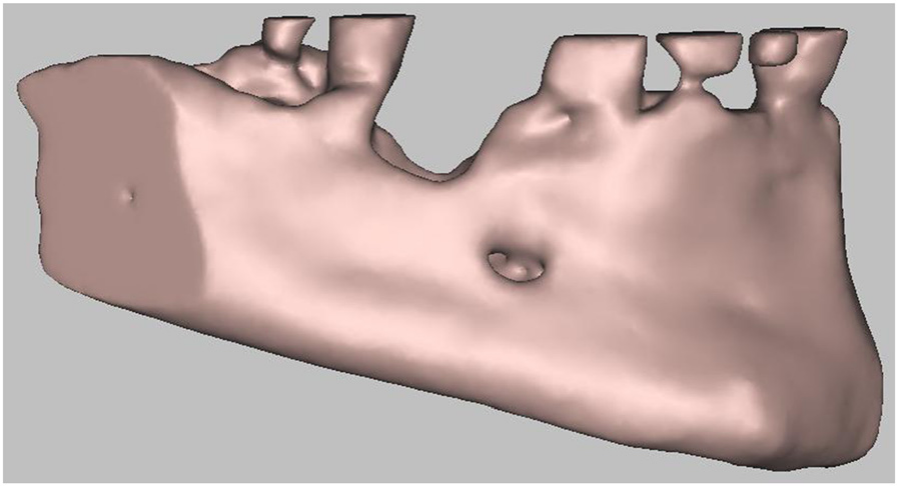
3D digital volumetric reconstruction of the mandibular defect

**Fig. 6:**
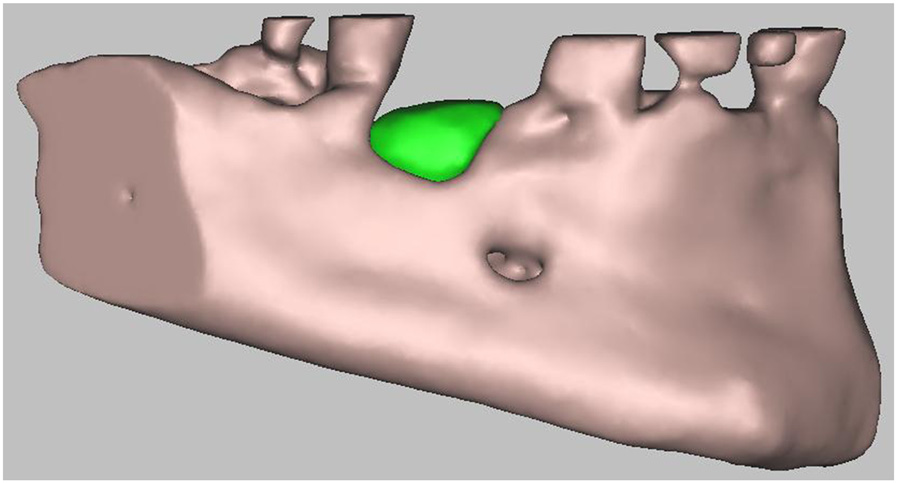
3D digital volumetric reconstruction of HA scaffold placed in the defect

**Fig. 7:**
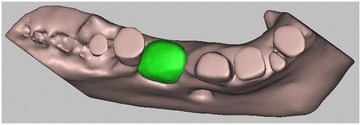
3D digital volumetric reconstruction of HA scaffold placed in the defect

**Fig. 8:**
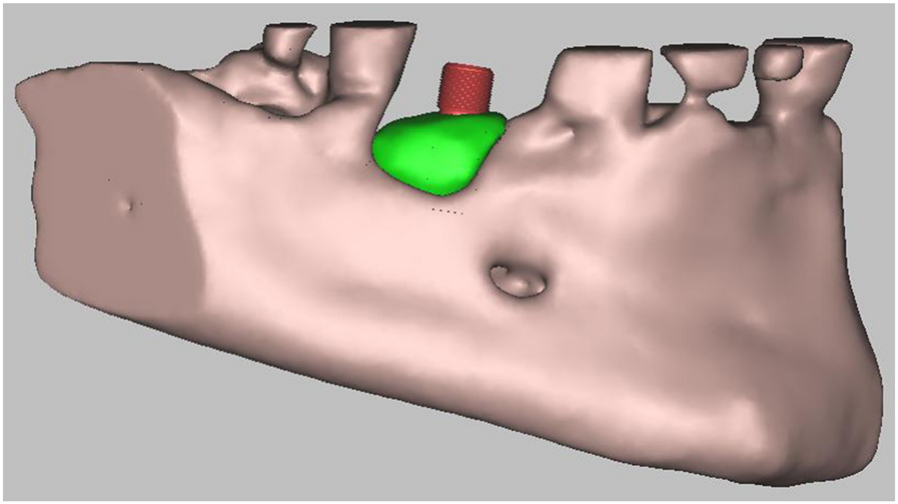
3D digital volumetric reconstruction of implant placement in regenerated bone

**Fig. 9:**
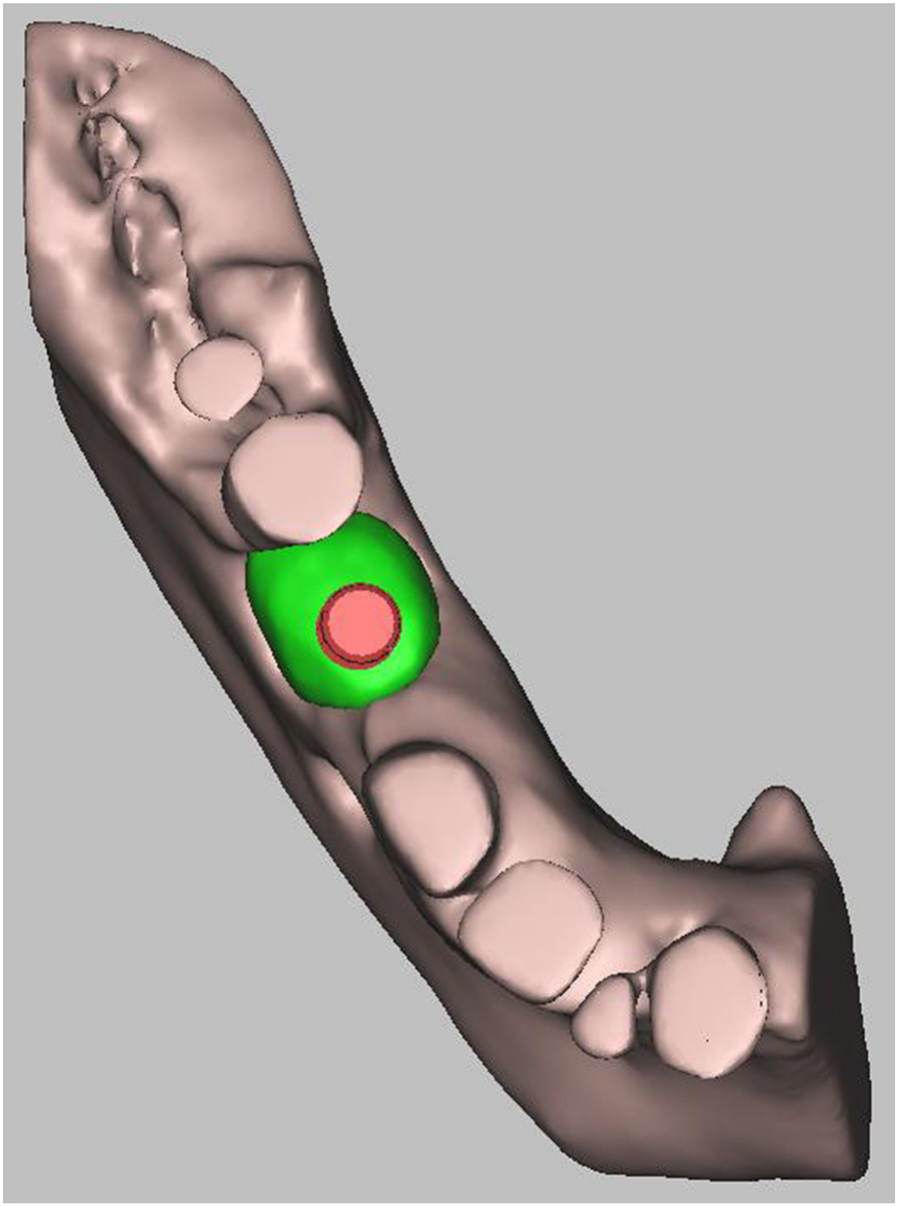
3D digital volumetric reconstruction of implant placement in regenerated bone

**Fig. 10:**
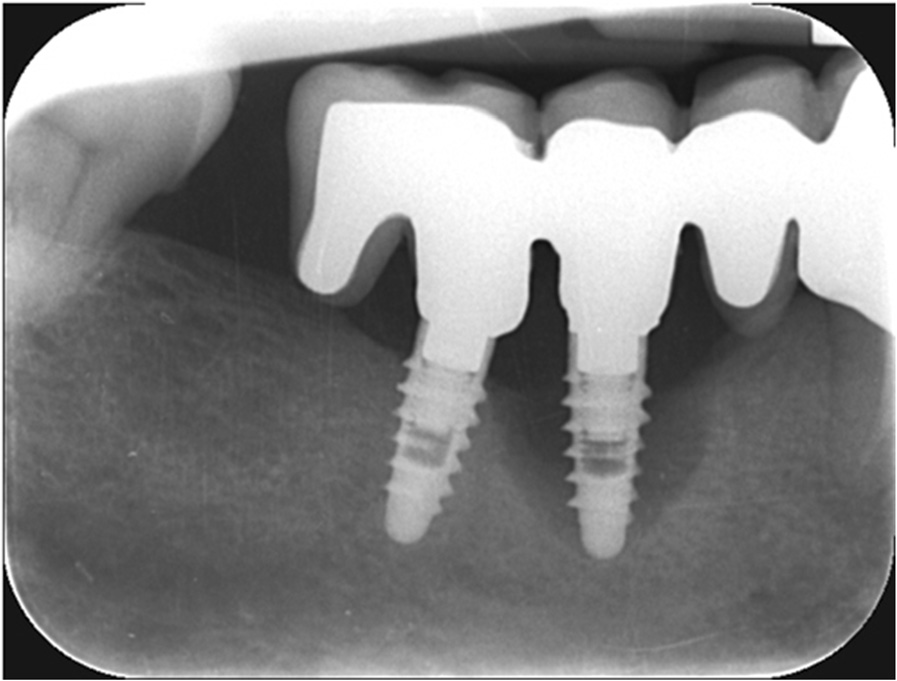
Intraoral radiography: the peri-implant defect

**Fig. 11:**
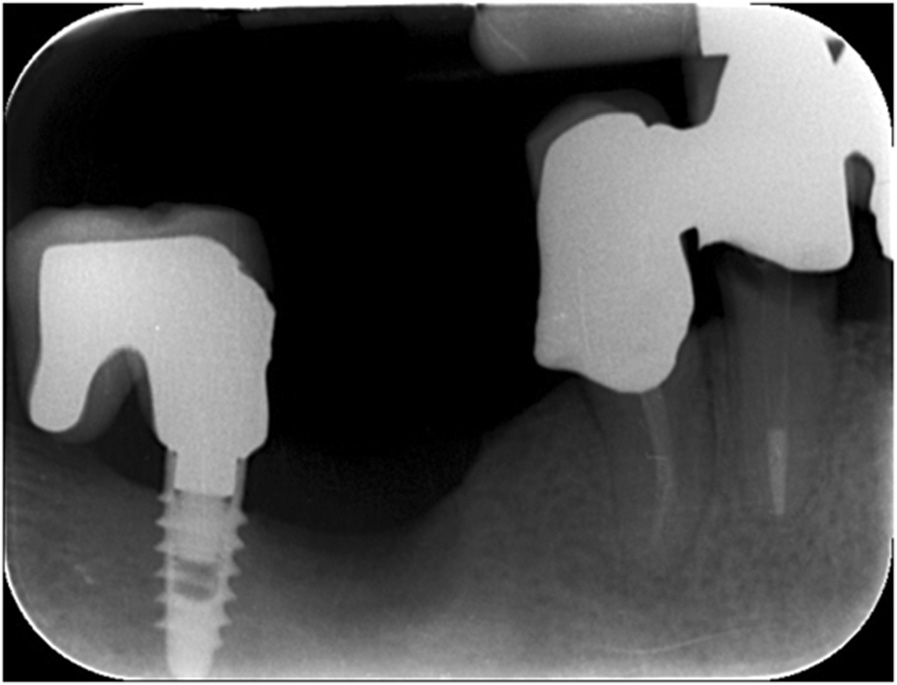
Intraoral radiography: defect after implant removal

**Fig. 12:**
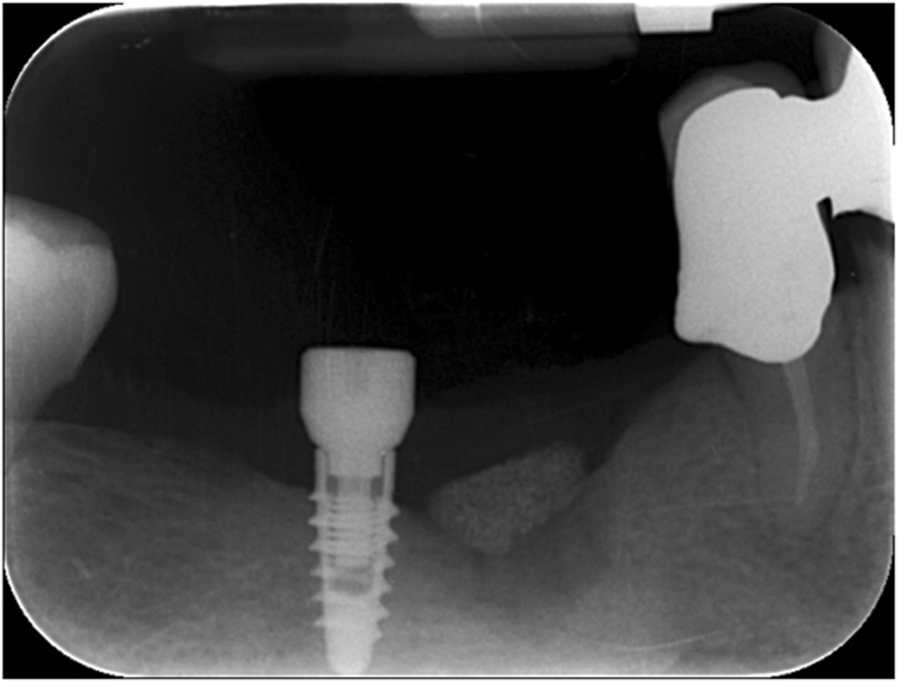
Intraoral radiography: HA scaffold placement

**Fig. 13:**
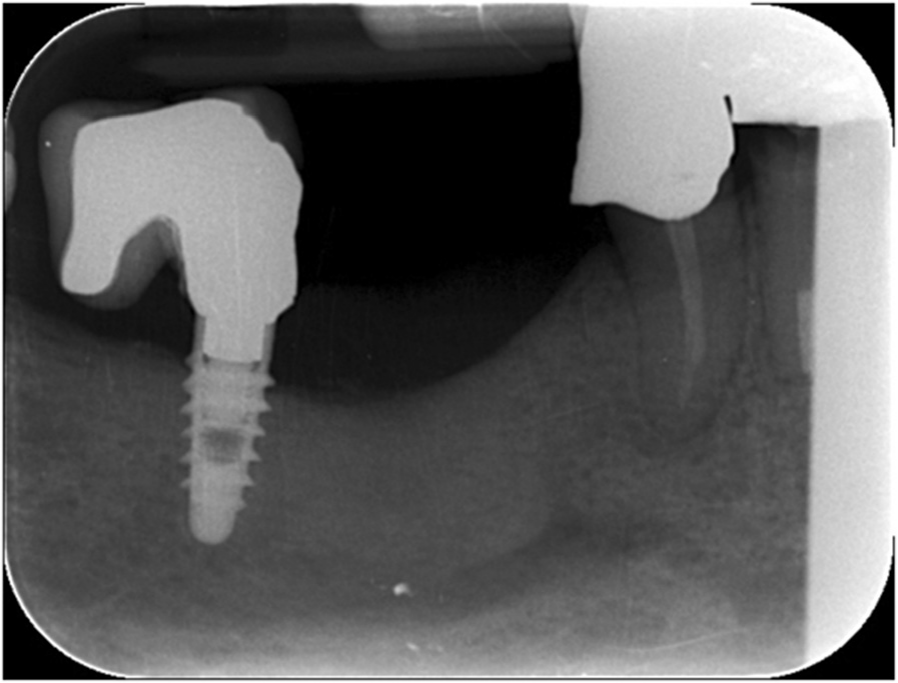
Intraoral radiography: regenerated bone after 5 months

**Fig. 14:**
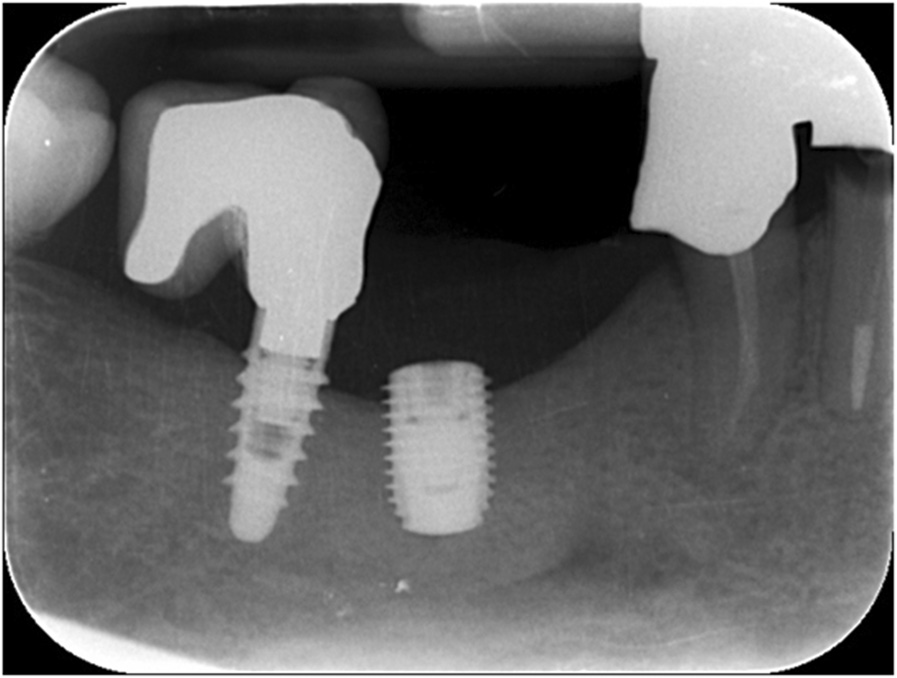
Intraoral radiography: implant placement

**Fig. 15:**
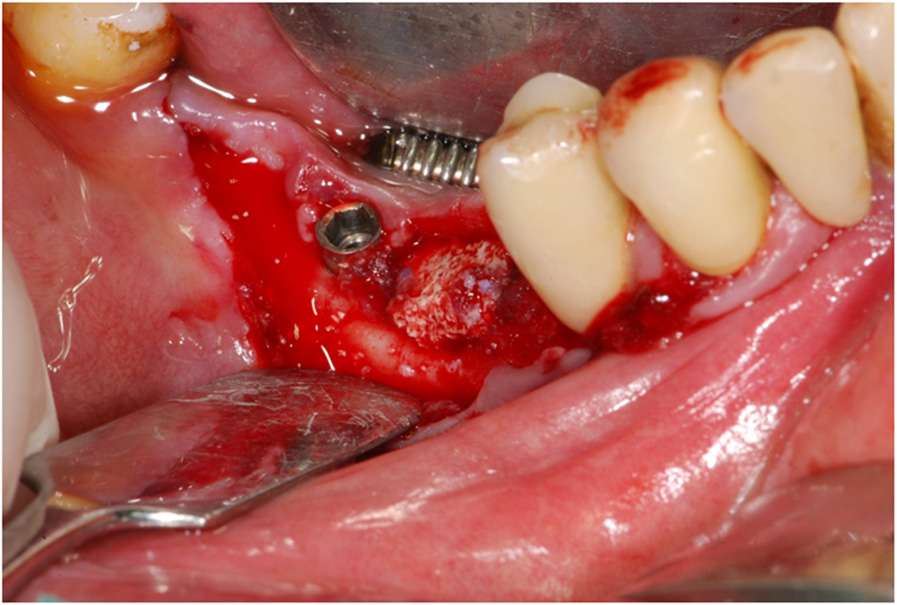
Clinical view: HA scaffold placement

**Fig. 16:**
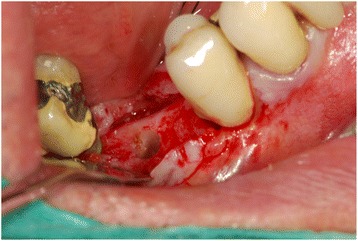
Clinical view: regenerated bone after 5 months

**Fig. 17:**
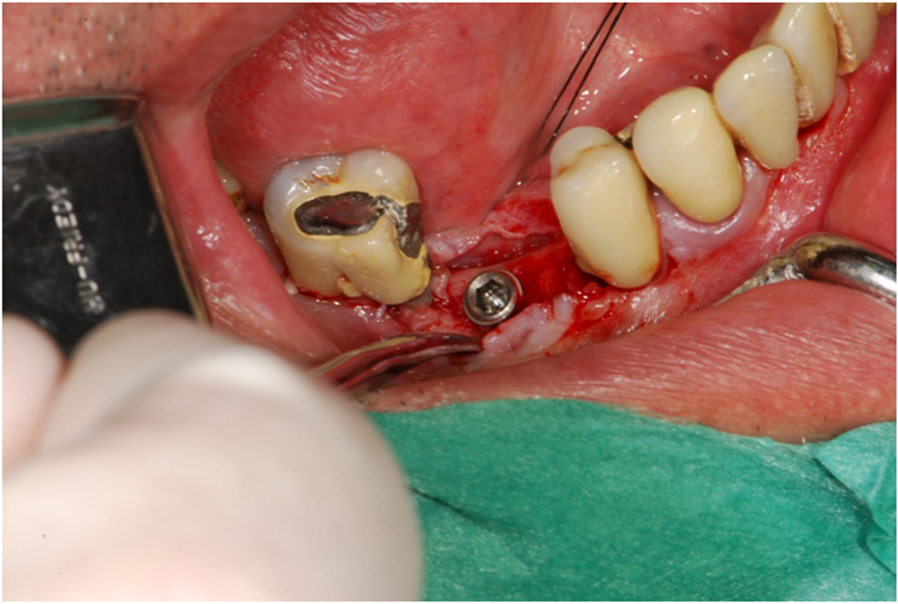
Clinical view: implant placement

A 55-year-old male presented with perimplantitis on one mandibular implant in the premolar area, requiring implant removal and bone regeneration procedures to allow new implant placement. The patient demonstrated good general health with no local or systemic contraindications to oral surgery or implant placement. Before the surgical procedure began, the patient received oral hygiene instructions and gave signed informed consent. Preoperative clinical and radiographical examinations were used to assess the morphology of the alveolar ridge (Figs. 5, 6, 7, 8, and 9). Presurgical medication of the patient consisted of a 0.2 % chlorhexidine gluconate mouth rinse for 2 min and an extraoral scrub with povidone-iodine solution. Local anesthesia consisted of 4 % articaine 1:100,000 and epinephrine. At 2 months, after the implant was removed (Figs. 10 and 11), a full thickness midcrestal incision was made and a mucoperiosteal flap was raised. Subsequently, a CAD/CAM hydroxyapatite scaffold was placed in the defect area, and the flap was replaced in the natural position. After a healing period of 5 months, integration of the hydroxyapatite into the newly formed bone was confirmed by CT exam and a radiographic control. The bone volume following regeneration was satisfactory, and one short osseointegrated implant (4.75 × 7 mm) was placed in position 45 (Figs. 12, 13, 14, 15, 16, and 17). After 3 months, a radiographic assessment showed successful integration between the implant and regenerated bone.

## Conclusions

Porous synthetic scaffolds have attracted considerable attention for applications in bone tissue engineering, because of the possibility of controlling every single feature (chemical composition, dimensions of the macropores, specific external morphology) to adapt the material to the specific clinical situation, especially when a sufficient quantity of autologous bone is not available. The possibility to project bone structures (using CAD/CAM techniques) could represent an additional advantage, compared to DBBM, to fit different clinical defects. Among the synthetic materials, HA has shown excellent mechanical features, osteoconductivity, and biocompatibility in vitro and in vivo compared to other biomaterials. A HA graft can be sterilized using gamma rays and is suitable for milling and finishing. It is then sterilized in an autoclave as in typical dental surgical procedures. To refine the material, appropriate drills have to be used because a mixture of HA to 60 % does not have the same rigidity as an autologous bone, but it does have sufficient rigidity to allow the milling and positioning of surgical screws. Porous HA is a more readily resorbable and more osteoconductive material than dense HA; however, its strength decreases exponentially with increased porosity. Mechanical tests have shown that fabricated HA scaffolds with pore diameters ranging from 400 to 1200 μ had compressive moduli and strengths within the range of the human craniofacial trabecular bone; this indicates that they can be used for bone regenerative rehabilitations. Several studies in vivo have evaluated optimal clinical and radiological results using HA scaffolds as grafts before implants are positioned after several months in both the human mandible and maxilla. In conclusion, using CAD/CAM techniques with HA scaffolds can increase graft stability and reduce surgical operating times. Today, digital computed tomography (CT) images, combined with CAD/CAM techniques, can be used as tools to directly produce customized devices in a biocompatible scaffold material, providing a valuable alternative to bone replacement based on autograft procedures. CAD/CAM technologies have enabled a new age in dentistry. The development of CAD/CAM software, implemented with radiology procedures, for the easy acquisition and transfer of DICOM 3 (Digital Imaging and Communications in Medicine) data, allows the surgeon to analyze the patient by performing three-dimensional measurements and to manipulate deformed or missing anatomy by segmentation and insertion of unaltered or idealized skeletal reconstructions. CAD/CAM technologies can be used in the field of maxillofacial bone reconstruction to improve the precision of treatment.

The current method consists of printing CT scan data to produce a three-dimensional stereolithographic model of the maxilla/mandible, on which, preoperatively, the graft material can be shaped manually. Furthermore, with recent improvements in computer technology, combined with advanced three-dimensional cutting machinery, it is now possible to directly cut a block of bone substitute into the most appropriate shape that has been designed preoperatively using a three-dimensional computer simulation [31]. The current protocol can be divided into three phases: virtual planning and design of the custom-made scaffold, manufacture of the custom-made scaffold, and reconstructive surgery. The protocol offers several benefits: (1) The virtual environment permits ideal preoperative planning, and the intraoperative time is not consumed by approximately and repeatedly modeling the scaffold to the native alveolar defect (as in conventional procedures) [32]; (2) The approach offers precise, anatomically fitting scaffolds, with the benefit of increased stability and reduced operative time; and (3) The technique allows the accurate reproduction of the patient’s maxillary contour, thereby reducing the quantity of graft material that is required compared to conventional bone augmentation techniques.

Unfortunately, CAD/CAM procedures still have some limitations. The first is dimensional and is related to the maximum size of the customized scaffold (about 12 mm height × 10 mm width). The second is related to the presence of metallic restorations next to the edentolous area; in fact, the presence of artifacts may complicate the CAD process and the custom-made scaffold design. Third, the scaffold is designed for placement in the defect area and is not to be in contact with residual roots. In particular, the scaffold is designed to be at least 2 mm from the adjacent teeth (residual roots). This distance is decided during the CAD process; however, the presence of narrow spaces and partial root exposure may represent a limitation of this technique [28].

## Consent

Written informed consent was obtained from the patient for the pubblication of this report.
